# Comparing Dorsal Tangential and Lateral Views of the Wrist for Detecting Dorsal Screw Penetration after Volar Plating of Distal Radius Fractures

**DOI:** 10.1155/2017/1402517

**Published:** 2017-07-31

**Authors:** Juan M. Giugale, Mitchell S. Fourman, Deidre L. Bielicka, John R. Fowler

**Affiliations:** ^1^Department of Orthopaedic Surgery, University of Pittsburgh Medical Center, Pittsburgh, PA, USA; ^2^Department of Orthopaedic Surgery, Rutgers-Robert Wood Johnson Medical Center, New Brunswick, NJ, USA

## Abstract

**Background:**

The dorsal tangential (DT) view has been shown to improve the detection of dorsal screw perforation during volar distal radius fracture fixation. Here we performed a cadaveric imaging survey study to evaluate if the DT view was uniformly beneficial for all screws.

**Methods:**

Standardized placement of fixed-angle volar distal radius plates was performed on two cadavers. Fluoroscopic images depicting variable screw perforation of each of the four screw holes on the plate were generated. A 46-image survey was distributed at a large academic medical center. Respondents were asked to answer if the screw was perforating through the dorsal cortex in each image. Statistical analysis was performed using Fisher's exact test. A *p* value < .05 was considered significant.

**Results:**

The DT view offered a significantly more reliable determination of dorsal screw penetration than traditional lateral imaging for the radial-most screw at all degrees of perforation and the middle two screws at 2 mm of perforation. Residents and attendings had more accurate screw readings overall using the DT view.

**Conclusions:**

The DT view is superior to traditional lateral imaging in the detection of small amounts of dorsal perforation of the radial-most three screws of a fixed-angle volar plate.

## 1. Introduction

Although volar plate fixation has become the gold standard in the treatment of unstable distal radius fractures, extensor tendon irritation and rupture are known complications of this procedure. The etiology of extensor rupture is believed to relate to dorsal screw prominence in the majority of cases [1, 2]. Intraoperative lateral fluoroscopic films are typically used to assess screw depth after insertion. However, the presence of Lister's tubercle makes the distal radial cortex convex, not flat. A previous anatomic study showed that the height of Lister's tubercle ranges from 1.4 to 6.6 mm in relation to the radial border of the radius [3]. This implies that the screws radial and ulnar to Lister's tubercle can be proud several millimeters and still appear to be within the dorsal cortex on lateral fluoroscopic films. Prior work also examined the third extensor compartment floor through a limited dorsal approach after volar fixation and found that 12.5% of patients had dorsal screw prominence not identified on lateral fluoroscopic films [4].

The dorsal tangential view has recently received attention as an intraoperative fluoroscopic imaging technique that may better detect dorsal radius screw penetration [2, 5–7]. It has been shown to have a sensitivity of 95% for a dorsal screw protrusion of 1.0 mm when evaluated by two fellowship-trained orthopedic surgeons [7]. However, this technique has not been tested on multiple groups with variable fellowship and degree of training backgrounds.

Here we performed a cadaveric experiment and related survey study to compare the relative efficacies of the lateral and dorsal tangential (DT) views in the identification of dorsal screw prominence across training backgrounds. We hypothesized that the DT view is more effective than the lateral view in the detection of dorsal screw prominence at all experience levels.

## 2. Methods

**Figure 1:**
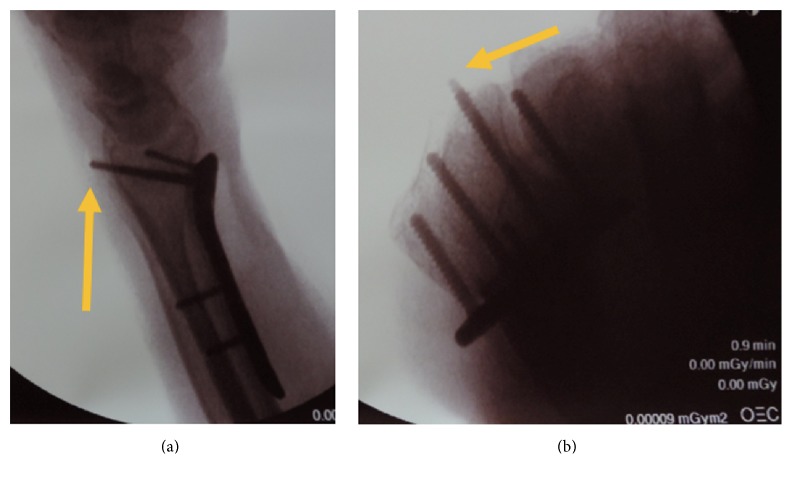
Fluoroscopic (a) dorsal tangential and (b) traditional lateral views of third screw from the radial aspect of the plate with a 4 mm dorsal cortex protrusion, as presented during survey analysis. Yellow arrows denote the protruding screw.

All protocol elements were approved by the Committee for Oversight of Research and Clinical Training Involving Descents (CORID #587) at our institution. All procedures followed were in accordance with the ethical standards of the responsible committee on human experimentation (institutional and national) and with the Helsinki Declaration of 1975, as revised in 2008. A traditional volar approach to the wrist was performed on two fresh-frozen cadaveric forearms, permitting the placement of a fixed-angle distal radius plate (Stryker, Kalamazoo, MI) with 4 distal screw holes. Screws were inserted into each of the distal holes with their tips flush with the dorsal cortex, confirmed by visual inspection through a separate dorsal incision. Plate positioning and placement were performed by the senior investigator (JRF). A screw from a single hole was then removed and replaced with a longer screw, producing a collection of images with standardized single screw prominences of 0, 2, 4, or 6 mm. Lateral and DT fluoroscopic radiographs were obtained after each screw exchange (Figures 1(a) and 1(b)). A true lateral was obtained by rotating the image until the pisiform overlapped the distal pole of the scaphoid. To produce the DT view, the wrist was maximally flexed and the forearm was placed at a 15° inclination in relation to the axis of the mini-C arm radiographic beam.

A total of 46 images were digitized into high quality digital image files and randomized using an online survey platform (Kwiksurveys, Bristol, United Kingdom). Surveys were distributed to orthopedic surgery residents, fellows, and faculty. Instructions for each survey question asked the respondent to consider each image individually and determine if the screw had penetrated the dorsal cortex. All questions were in a binary yes/no format and asked the respondent to identify any screw protrusion in each successive image. Results were analyzed with GraphPad Prism 6.0 (LaJolla, CA) using Fisher's exact test for categorical data, as normality cannot by assumed given the limited sample size included in this study. In all cases, significance was defined as *p* < .05.

## 3. Results

Completed responses were logged from a total of 18 respondents (7 faculty, 7 residents, and 4 fellows) out of a total of 52 faculty, fellows, and residents surveyed. A response was considered complete if it included answers to all questions. An additional five responses that failed to meet this criterion were removed from our sample size.

The overall accuracy of screw prominence across all-comers was significantly greater with the DT view (78.6%) compared to the lateral view (62.4%, *p* < .0001). Significant differences between DT and lateral view accuracy were also observed in the attending (80.3% versus 65.6%, *p* < .05) and resident (80.2% versus 57.6%, *p* < .0001) subgroups (Table 1). The composite sensitivity, specificity, and positive/negative predictive values of both groups were equivocal (Table 2).

The lateral view was unable to accurately identify 2 mm prominence at all screw positions (min accuracy 5.9 ± 8.3% at radial styloid screw versus 61.1% at ulnar corner screw) and continued to be problematic at all screw prominence lengths at the radial styloid view (13 ± 1.8% at 4 mm and 49.4 ± 61.5% at 6 mm). However, more ulnar positions showed improved accuracy (Table 3). In contrast, the DT view had significantly stronger performance at the radial styloid screw at all screw prominences (78.1 ± 4.4% at 2 mm, 93.5 ± 6.3% at 4 mm, and 96.9 ± 4.4% at 6 mm). The worst accuracy in the DT group was in identifying a 2 mm prominence of the ulnar corner screw (50 ± 44.2%). Statistical comparison between views at each screw position and prominence could not be performed because of the reduced number of total views available.

## 4. Discussion

Extensor tendon rupture after volar plate fixation of distal radius fractures may occur secondary to screw prominence. The protuberance of Lister's tubercle can mask dorsal screw penetration on lateral fluoroscopic views. The dorsal tangential (DT) view has been proposed as a method that can more sensitively detect dorsal screw prominence compared to standard lateral radiographs. Our work suggests that the primary advantage of the DT view compared to traditional lateral fluoroscopic views is in the evaluation of small degrees (2 mm) of screw protrusion on the radial three screws on a fixed-angle volar plate. Larger magnitudes of screw protrusion further limit the benefit of the DT view to the single most-radial screw on the plate. Overall, the DT view was found to be more reliable than the lateral view by all experience cohorts but active clinical fellows. However, the sensitivity and specificity of both techniques were still equivocal.

Our findings appear to stand in contrast to existing work. Ozer et al. [7] compared four radiographic views (lateral, 45 degree supination, 45 degree pronation, and dorsal tangential views) to determine the most sensitive view to detect screw protrusion. The authors found that the dorsal tangential view was sensitive at detecting screw penetration into the 3rd and 4th compartment (both compartments ulnar to Listers tubercle) with a sensitivity of 88–98% for screws 1–3 mm proud. However, the sensitivity of the dorsal tangential view to 1–3 mm proud screws radial to Lister's tubercle was only 35–48%. In contrast, the results of this study noted an increased accuracy of the DT view compared to the lateral view in the evaluation of more radial screws (Table 3), while ulnar imaging was generally equivocal between both views.

This disagreement may be independent of the benefit of the DT view. Cadaveric work by Perry et al. [8] found that the central and slightly ulnar screws of more commonly used volar plate models were most likely to include the extensor pollicis longus tendon in their k-wire and screw trajectory. In an evaluation of 18 cadaveric arms, none of the most-radial or most-ulnar screw holes were associated with EPL interaction and were therefore not labeled by that study as “high risk.” Further, the “at risk” central screws were also found to be more likely to penetrate their corresponding extensor compartments, a finding supported by other cadaveric and ultrasound studies [9, 10]. In our work, the DT view was superior to the lateral view in the identification of a 2 mm prominence of the two central screws of the plate used in our model. This small prominence has still been associated with tendon pathology, particularly in those screws that threaten the third or fourth dorsal compartments [10]. Based on this additional evidence, the benefit of the DT versus lateral view at the radial- or ulnar-most screw holes of any plate appears to be clinically irrelevant. Instead, the improved sensitivity of the DT view in the identification of a small amount of dorsal cortex perforation through central plate holes highlights its true clinical benefit.

While Wall et al. [11] demonstrated that unicortical distal radius screws spanning 75% of the bone offer a similar biomechanical construct to bicortical screw fixation, coronal shear injuries require bicortical fixation to optimize construct rigidity. In any fracture pattern that requires bicortical fixation, vigilance should be exercised to avoid dorsal screw prominence and the may be considered to ensure minimal screw penetration. Joseph and Harvey [12] used the DT view as a final fluoroscopic check prior to skin closure in 15 distal radius fractures with a coronal-split element and found that the DT view led to screw exchange in 26.6% of cases. In general, the DT view has been found to be a better “rule-out” indicator than “rule-in,” with a negative predictive value of 97% but a sensitivity of only 67% [13]. Additional benefits of the DT view include reduced radiation exposure during evaluation, which is beneficial if using this technique for screw exchange prior to closure [14], as well as confirmation of adequate reduction of the sigmoid notch during fixation [15].

While the findings of this work appear to be largely supported by prior work, our study has several limitations. There is a small amount of unpreventable variability in positioning and obtaining the dorsal tangential and lateral fluoroscopic films. As demonstrated by Haug et al. [6], the accuracy of the dorsal tangential view decreases as inclination of the forearm deviates from 15° relative to the axis of the fluoroscopic beam. We accepted this slight variability, recognizing that a similar deviation from optimal beam position would exist clinically. The aim of our cadaveric study was to better understand the utility of two different fluoroscopic views in identifying screw penetration. The true clinical impact of each screw is likely not to be equal and cannot be quantified by our study. It is therefore reasonable to postulate that variability between these two imaging techniques may not translate into a similar degree of difference clinically. This is important when considering the slight variability that may exist between plate manufacturers, although our findings suggest that radially placed holes, wherever their exact position, will still benefit from imaging from the dorsal tangential view. Further investigation is required to determine if the dorsal tangential view affects the rate of extensor tendon rupture clinically and if this complication leads to worse clinical outcomes. We must also assess intraobserver consistency to confirm that our findings are reproducible.

## 5. Conclusion

In a survey study based on measurements made using a cadaveric model, we found that the DT view is superior to the lateral view of the wrist in the identification of any degree of dorsal cortex perforation of the radial-most screw in a volar locking plate. Further, the DT view may also be superior to the lateral view in the detection of 2 mm screw prominence in central plate holes. Further work that utilizes an increased sample size and image quantity and considers additional plate types and hole configurations is required to validate the findings of this pilot work.

## Figures and Tables

**Table 1:** Cohort and subgroup accuracy in the identification of screw prominence using either the DT or lateral views.

	Accuracy DT view	Accuracy lateral view	*p* value
Cohort (*n* = 18)	78.6%	62.4%	<.0001^*∗*^
Attending (*n* = 7)	80.3%	65.6%	<.05^*∗*^
Fellow (*n* = 4)	73.2%	65.8%	.39
Resident (*n* = 7)	80.2%	57.6%	<.0001^*∗*^

^*∗*^Statistical significance (*p* < .05).

**Table:** (a) Lateral view

		Actual screw prominence	
		Prominent	Not prominent

Survey response	Prominent	212	128
	Not prominent	8	22
		Sensitivity	96.4%
		Specificity	14.7%
		PPV	62.4%
		NPV	73.3%

**Table:** (b) DT view

		Actual screw prominence	
		Prominent	Not prominent

Survey response	Prominent	274	71
	Not prominent	8	21
		Sensitivity	97.2%
		Specificity	22.8%
		PPV	79.4%
		NPV	72.4%

**Table 3:** Overall accuracy based on imaging technique and screw position and degree of prominence.

	1 (radial styloid)	2	3	4 (ulnar corner)
DT 0 mm	84.6% (1 image)			
Lat 0 mm	85.7% (1 image)			
DT 2 mm	78.1 ± 4.4%	100% (1 image)	79.5 ± 11.4%	50 ± 44.2%
Lat 2 mm	5.9 ± 8.3%	35.6 ± 49.01%	18.9% (1 image)	61.1% (1 image)
DT 4 mm	93.45 ± 6.3%	100% (1 image)	60 ± 56.6%	65.8 ± 40.0%
Lat 4 mm	13.0 ± 1.8%	75 ± 35.4%	100% (1 image)	83.3% (1 image)
DT 6 mm	96.9 ± 4.4%	100% (1 image)	75.2 ± 26.8%	78.9 ± 3.4%
Lat 6 mm	49.4 ± 61.5%	97.6 ± 4.1%	100% (1 image)	50.8 ± 61.7%
